# Hydrophilic Polymers Enable Gastrointestinal Transit Monitoring by Fluorine‐19 Magnetic Resonance Imaging

**DOI:** 10.1002/adhm.71667

**Published:** 2026-09-13

**Authors:** Ondřej Groborz, Vít Herynek, Mária Hovořáková, Zuzana Pavlíková, Linda Dalecká, Klára Steklíková, Petr Matouš, Petr Páral, Margarita Tkachenko, Filip Koucký, Fatma Nalan Cetin, Ludmila Maffei Svobodová, Jiří Beneš, Kristýna Kolouchová

**Affiliations:** ^1^ Institute of Organic Chemistry and Biochemistry Czech Academy of Sciences Prague Czech Republic; ^2^ Institute of Biophysics and Informatics First Faculty of Medicine Charles University Prague Czech Republic; ^3^ Center For Advanced Preclinical Imaging (CAPI) First Faculty of Medicine Charles University Prague Czech Republic; ^4^ Institute of Histology and Embryology First Faculty of Medicine Charles University Prague Czech Republic; ^5^ Institute of Pathophysiology First Faculty of Medicine Charles University Prague Czech Republic; ^6^ Department of Inorganic Chemistry Faculty of Science Charles University Prague Czech Republic; ^7^ Polymer Chemistry and Biomaterials Group Centre of Macromolecular Chemistry Department of Organic and Macromolecular Chemistry Ghent University Ghent Belgium; ^8^ Faculty of Health Studies Technical University of Liberec Liberec Czech Republic

**Keywords:** ^19^F MRI, gastrointestinal motility, noninvasive, poly [N‐(2,2‐difluoroethyl)acrylamide], poly [N‐(2‐hydroxypropyl)methacrylamide], tracer

## Abstract

Gastrointestinal (GI) transit shapes nutrient absorption and oral drug delivery. However, GI anatomy, function, and motility disorders are primarily examined by radiographic imaging, which exposes patients to ionizing radiation and yields only coarse proxies of how a meal moves through the gut. By contrast, fluorine‐19 (^19^F) MRI offers background‐free, quantitative tracking, but most oral ^19^F agents are hydrophobic and phase‐separate from chyme, generating layering artifacts, which spoil motility and anatomy readouts. Here, we developed nonresorbable, hydrophilic fluorinated polymer tracers miscible with luminal contents. These tracers provided a strong, quantifiable ^19^F signal for combined ^1^H/^19^F MRI and ^19^F spectroscopy, enabling us to visualize GI anatomy and transit noninvasively. In vivo imaging reliably tracked whole‐gut transit and fecal elimination, with no evidence of systemic uptake or tissue injury. As such, this practical, reproducible, and radiation‐free platform for phenotyping dysmotility and monitoring therapeutic response may open up opportunities for longitudinal GI studies and clinical trials.

## Graphical abstract

Hydrophilic fluorinated copolymers dissolve in chyme and give a strong, single ^1^ ^9^F signal, unlike phase‐separating perfluorocarbons. Combined ^1^H‐^1^ ^9^F MRI tracks their whole‐gut transit non‐invasively and without ionizing radiation. In rats, the tracers move through the gastrointestinal tract, resist resorption, and are well tolerated, a proof‐of‐concept for radiation‐free monitoring of gut motility.

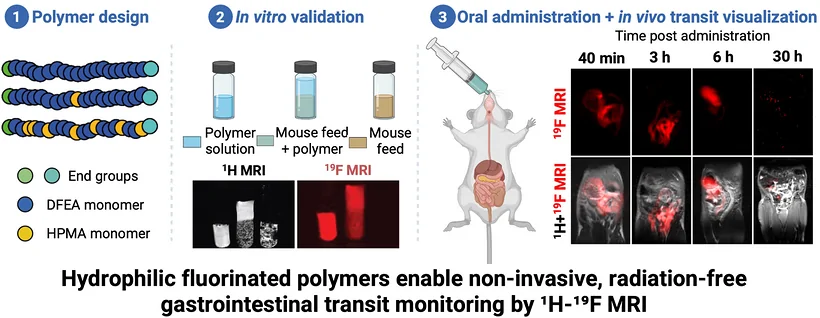

## 1 Introduction

Gastrointestinal tract (GIT) transit impacts nutrient absorption [1, 2], medication efficacy [3, 4, 5], and overall metabolic balance [1, 6]. In fact, abnormal transit has high prevalence rates in both organic and functional gastrointestinal disorders [7, 8, 9]. To examine GIT transit and anatomy, physicians rely on noninvasive imaging methods, such as ultrasound [10], scintigraphy [11, 12, 13, 14], and radiography using contrast agents [11, 14]. However, these methods entail several downsides. Ultrasound reveals only a small GIT segment at a time, poorly tracks the chyme, and provides no GIT transit time data [10]. Scintigraphy is lengthy and costly, requiring advanced hardware and training, in addition to exposing both patients and medical staff to ionizing radiation [15]. And while radiographic techniques can provide valuable information about GIT function, the contrast agents may cause severe side effects [16]. Simultaneously, these methods expose patients to high levels of ionizing radiation, ranging from 5 to 50 mGy [17, 18, 19], discouraging their use, especially in pregnant women and children [19, 20, 21]. In this context, further research efforts must be made to develop alternative imaging methods [22].

Magnetic resonance imaging (MRI) may not be commonly used in gastroenterology yet, but is a highly promising method nonetheless, without ionizing radiation or a limit on penetration depth. Case in point, MRI has been applied to study anatomical structures [23] and motor functions [14, 24, 25] of the GIT, and the US Food and Drug Administration (FDA) has already approved perfluorooctyl bromide as a negative contrast agent for GIT imaging for clinical practice [26]. Moreover, the scope of MRI applications can be further extended with only minor hardware and software modifications [27], as shown by fluorine‐19 MRI (^19^F MRI) [28, 29, 30].

^19^F MRI can be performed with minute ^19^F concentrations. Under laboratory conditions, ^19^F MRI can detect ≥ 2·10^13^ fluorine atoms. In clinical practice, concentrations in the range of mmol/L ^19^F atoms may be needed for human imaging in common 3T scanners [31]. As the only naturally occurring, nonradioactive nuclide of fluorine, ^19^F reaches up to 83% MR receptivity of ^1^H [32]. Because ^19^F content in the body is negligible [33, 34], ^19^F MRI detects only xenobiotic fluorinated tracers, without background [32]. Furthermore, several tracers can be measured thanks to the wide range of ^19^F chemical shifts (over 300 ppm) [35], and ^19^F MR images can be acquired on ordinary ^1^H MRI scanners with ^1^H/^19^F MR radiofrequency coils [27]. In some setups, the scanner can even acquire ^1^H + ^19^F MR images simultaneously [36], highlighting anatomical structures and the precise location of the tracer. Accordingly, coupling ^1^H+^19^F MRI with a nonresorbable fluorinated tracer may provide a noninvasive, ionizing radiation‐free method for monitoring GIT function.

For GIT ^1^H+^19^F MRI, biologically inert, nonresorbable perfluorocarbons have been tested as tracers [28, 29, 30, 37, 38]. However, most perfluorocarbons lack suitable ^19^F MR properties [32]. Their multiple signals and poor MR relaxation properties (*T*_1_ ≈ 1100 ms, *T*_2_ ≈ 10 for CF_2_, and *T*_2_ ≈ 290 ms for CF_3_) [39, 40, 41] complicate imaging. Making matters worse, these molecules are highly hydrophobic and do not mix well with chyme, inducing imaging artifacts [32] and precluding reliable GIT transit imaging. Although the latter issue can be compensated with emulsion nanocarriers that improve perfluorocarbons’ dispersibility [79], doing so usually deteriorates the ^19^F MR relaxation properties [80, 81]. Moreover, such systems require surfactants, are difficult to prepare, and their stability under GIT conditions (gastric acid, bile salts, peristaltic shear) [82] over multi‐hour transit may be an issue. Nevertheless, fluorinated hydrophilic polymers, such as poly [*N*‐(2,2‐difluoroethyl)acrylamide] (PDFEA) and its copolymers, may be used to develop ^19^F MRI tracers for GIT transit, leveraging their (i) high fluorine content (28 wt. %), (ii) low toxicity, and (iii) near‐optimal MR properties (a single signal with *T*_1_ ≈ 400 ms and *T*_2_ ≈ 150 ms) [42]. Therefore, fluorinated hydrophilic polymers may yield nonresorbable tracers for noninvasive GIT transit examination.

In this study, we aim to test DFEA (co)polymers as new tracers for GIT transit imaging. For this purpose, we synthesized three polymers, that is, a **PDFEA_100_** homopolymer and its two copolymers with poly(hydroxypropyl)methacrylamide (HPMA), more specifically **P(DFEA_94_**‐***co*‐HPMA_6_**) and **P(DFEA_63_**‐***co*‐HPMA_37_**). We tested various amounts of monomers [43] to maximize fluorine content while suppressing thermoresponsiveness to mitigate polymer aggregation‐induced loss of ^19^F MRI signal [44, 45]. Aqueous solutions of these polymers provided strong and sharp ^19^F MR signals, but unlike perfluorocarbons, these polymers mixed well with mouse feed. After injecting these polymers into rat stomachs, we were able to noninvasively observe GIT transit. Moreover, our histopathological analysis revealed no signs of toxicity in these animals. These findings highlight the potential of hydrophilic fluorinated polymers to become next‐generation tracers for GIT transit monitoring by ^19^F MRI.

## 2 Results

We synthesized three DFEA‐based polymers with similar molar masses, namely a homopolymer (**PDFEA_100_**) and two statistical copolymers (**P(DFEA_94_‐*co*‐HPMA_6_)** and **P(DFEA_63_‐*co*‐HPMA_37_)**), via reversible addition‐fragmentation chain transfer (RAFT) polymerization (Figure 1A,B). Their number‐average molar mass ranged from 50 to 60 kg/mol, with a narrow molar mass dispersity (*Ð*_M_ ≈ 1.1 to 1.2), as shown by gel permeation chromatography. By ^1^H NMR spectroscopy, we confirmed that HPMA was incorporated into the copolymers, with matching monomer ratios. For ex vivo fluorescence imaging of the polymers, their terminal carboxylic acid groups were quantitatively labelled with a fluorescein derivative.

**FIGURE 1:**
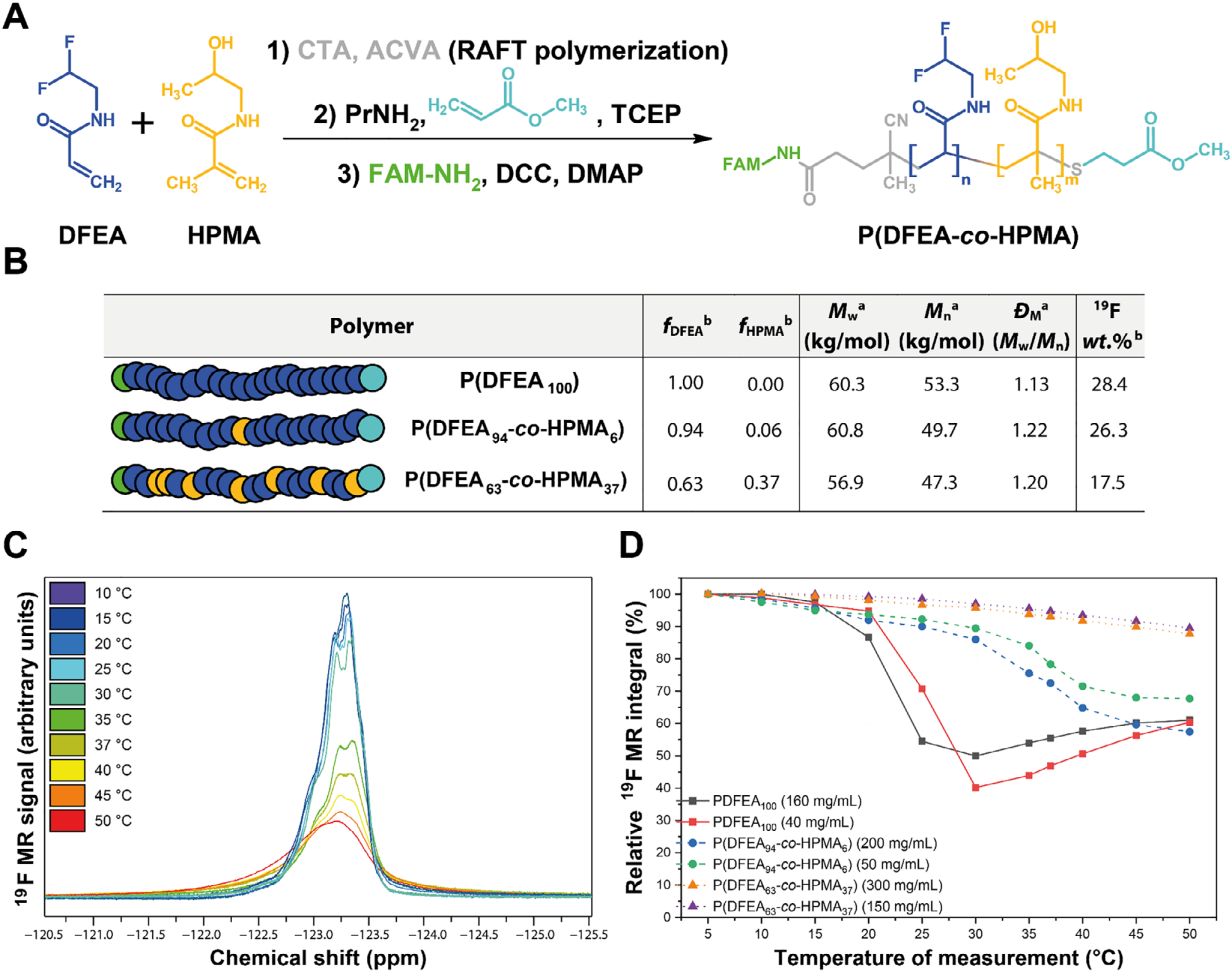
Increasing polymer hydrophilicity (HPMA content) prevents the loss of ^19^F MR signal (at 9.4 T) at higher temperatures and polymer concentrations. (A) DFEA copolymerization with HPMA via RAFT and condensation of the terminal carboxylic moiety with fluorescein‐amine (FAM‐NH_2_). (B) Scheme of three DFEA polymers differing in HPMA content and their physicochemical properties; *f*_DFEA_ and *f*_HPMA_ stand for the molar fraction of a specific monomer within an average polymer chain; *M*_w_, for mass‐average molar mass; *M*_n_, for number‐average molar mass of the polymer; and *Ð*_M_, for dispersity index; ^19^F wt. %, for the percentage of fluorine atoms (per weight) in the dry (co)polymer. Values determined by ^a^GPC (Figure S7) and by ^b^GPC+NMR (Figures S9 and S10). (C) ^19^F MR spectrum of an aqueous solution of **P(DFEA_94_‐*co*‐HPMA_6_)** showing loss of signal with the increase in temperature due to polymer aggregation (thermoresponsiveness). (D) Variation of relative ^19^F signal as a function of temperature and polymer concentration.

### 2.1 HPMA Incorporation Increases Polymer Solubility in Water and Fluorine Concentration

Since ^19^F MR imaging of GIT transit requires a high concentration of fluorine atoms in solution, we tested the solubility of our compounds in water. The homopolymer **PDFEA_100_** dissolved in cold water over several days, and its concentration never exceeded 160 mg/mL, aggregating even at 4°C due to its thermoresponsiveness. As a result, the maximum concentration of fluorine atoms in solution was 4.7 wt. %. In contrast, **P(DFEA_94_‐*co*‐HPMA_6_)** and **P(DFEA_63_‐*co*‐HPMA_37_)** copolymers were much more soluble, with concentrations ≥ 200 mg/mL and without aggregation at room temperature (Figure 1D). Despite having lower fluorine content per gram of polymer (Figure 1B), **P(DFEA_94_‐*co*‐HPMA_6_)** and **P(DFEA_63_‐*co*‐HPMA_37_)** reached higher overall concentrations of fluorine atoms in solution (Table S8). Thus, these statistical copolymers should be more suitable for ^19^F MRI GIT tracing than **PDFEA_100_**.

### 2.2 HPMA Prevents Aggregation‐Induced ^19^F, MR Signal Loss

At body temperature, **PDFEA_100_** solutions aggregate and lose ≈ 50% of their MR signal (Figure 1C,D), because aggregated polymer has fast *T*_2_ relaxations and is therefore invisible to MR [44, 45]. Subsequently, the ^19^F MR signal increased slightly at higher temperatures, most likely upon polymer self‐assembly into nanoparticles [46]. The ^19^F MR signal of **P(DFEA_94_‐*co*‐HPMA_6_)** and **P(DFEA_63_‐*co*‐HPMA_37_)** also weakened with temperature, but much less so than that of **PDFEA_100_** and only at higher temperatures (Figure 1D). At 37°C, **P(DFEA_94_‐*co*‐HPMA_6_)** and **P(DFEA_63_‐*co*‐HPMA_37_)** solutions lost ≈ 25% and less than 10% of their MR signal, respectively. These results confirm that incorporating a hydrophilic co‐monomer into PDFEA increases the temperature of aggregation, that is, the cloud point temperature (*T*_CP_) [43]. Accordingly, HPMA incorporation not only increases polymer solubility and ^19^F concentration in solution but also prevents ^19^F MR signal loss caused by polymer aggregation.

### 2.3 Hydrophilic Polymers Show Suitable Properties for ^19^F MR Imaging

Neat (solvent‐free) perfluorooctane contains four chemically non‐equivalent populations of fluorine atoms with markedly different ^19^F MR relaxation times (Table S8). At 9.4 T, all fluorine atoms had long *T*_1_ relaxation times (≈ 950 to 1700 ms). In turn, the *T*_2_ relaxation times varied among individual moieties: CF_3_ had a very long *T*_2_ (≈ 550 to 700 ms), but CF_2_ had a relatively short *T*_2_ (≈ 20 to 50 ms). The relaxation properties of neat perfluorooctane are not available in the literature, but analogous neat perfluorocarbons (perfluorooctyl bromide at 3T [40] and perfluorononane at 9.4 T [39] have similar ^19^F MR properties (multiple signals; *T*_1_ ≈ 1100 ms, *T*_2_ ≈ 10 for CF_2_ and *T*_2_ ≈ 290 ms for CF_3_). Moreover, perfluorooctane provided multiple ^19^F MR signals over the span of 37 ppm, so (a) only some of its ^19^F MR signals can be used for imaging, and (b) perfluorooctane samples tend to cause chemical‐shift MRI artifacts [32]. Consequently, only a minority of fluorine atoms in perfluooroctane are suitable for ^19^F MR imaging (only 26 wt. % of the mass of fluorine).

In sharp contrast to perfluooroctane samples, aqueous solutions of **PDFEA_100_**, **P(DFEA_94_‐*co*‐HPMA_6_)**, and **P(DFEA_63_‐*co*‐HPMA_37_)** showed only one MR peak around –126.5 ppm, with a single population of *T*_1_ relaxation times around 350 ms at 20°C and from 400 to 450 ms at 37°C, but with two populations of *T*_2_ relaxation times. At 20°C, the fast‐relaxing population had a *T*_2_ from 5 to 10 ms and the slow‐relaxing *T*_2_ ≈ 100 to 200 ms. In turn, at 37°C, both relaxation times usually increased from 40 to 70 ms and 200 to 270 ms, respectively, corroborating our previous studies [42, 47]. In short, PDFEA copolymers have much more suitable *T*_1_, *T*_2_, and *T*_2_‐to‐*T*_1_ ratios for ^19^F MR imaging than perfluorocarbons, and their relaxation times are affected by temperature, but not polymer concentration.

### 2.4 Hydrophilic Polymers Provide a Uniform ^19^F MR Signal Throughout Chyme

To assess the ability of fluorinated tracers to mix with feed, we homogenized rodent feed into a powder (individual pieces ≤ 2 mm, Figure S4) and mixed this powder with water to mimic chyme. We placed 1 mL of this chyme model into a small vial with either 1 mL of polymer solution or 1 mL of perfluorooctane and mixed them thoroughly before acquiring ^1^H and ^19^F MR images (Figure 2). Shortly after mixing, the dense and hydrophobic perfluorooctane sank to the bottom of the vial (Figure S5b, Figure 2A), resembling an intraluminal layering artifact [32]. Since fluorocarbons are poorly soluble in the water layer [48] and vice‐versa [49], the perfluorooctane layer did not produce a detectable ^1^H MR signal (indicating minimal water content), and the chyme layer did not produce a detectable ^19^F MR signal (indicating minimal perfluorooctane content). Consequently, the ^1^H MR image showed only the chyme, and the ^19^F MR image showed only the layer of perfluorooctane (Figure 2B,C). In contrast, the aqueous PDFEA solution (160 mg/mL of **PDFEA_100_**; density 1.043 ± 0.005 g/cm^3^ at 15°C) remained mixed with the chyme. For this reason, we detected strong ^1^H and ^19^F MR signals throughout the sample (Figure 2), demonstrating that hydrophilic polymers are well‐suited for chyme tracking.

**FIGURE 2:**
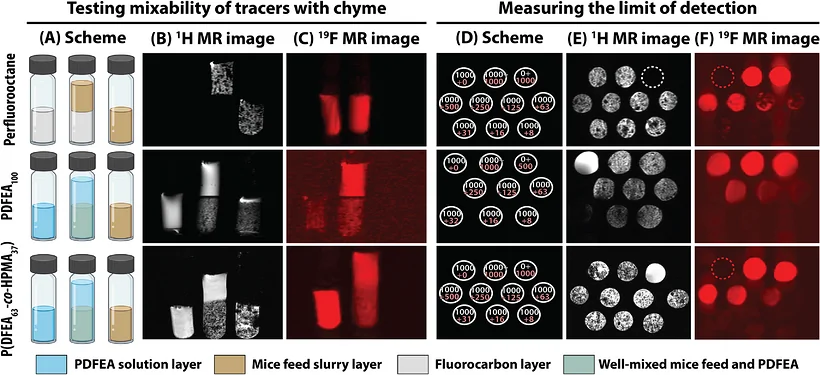
**PDFEA_100_** and **P(DFEA_63_‐*co*‐HPMA_37_)** solutions mix well with chyme, yielding a uniform fluorine signal throughout the phantom, unlike perfluorooctane. **LEFT**: Mixability tests of rodent feed slurry (chyme model) with perfluorooctane, **PDFEA_100_** (16 wt. %), and **P(DFEA_63_‐*co*‐HPMA_37_)** (25 wt. %): (**A**) experimental setup; three vials contained either fluorinated tracer, fluorinated tracer + chyme model, or only chyme. All samples were thoroughly mixed by shaking and vortexing. Nevertheless, perfluorooctane separated into two layers within 1 min, whereas all PDFEA samples remained homogeneous, as shown by (**B**) ^1^H and (**C**) ^19^F MR imaging. **RIGHT**: Comparison of in vitro detectability of phantoms with ^19^F MRI tracers: (**D**) an arrangement of phantom vials containing various amounts of chyme model (upper number, mg) and perfluorooctane, **PDFEA_100_** solution (16 wt. %) or **P(DFEA_63_‐*co*‐HPMA_37_)** (25 wt. %) (lower number, µL); (**E**) ^1^H and (**F**) ^19^F MR imaging of the phantoms. See Section S2.3.2 for further details. For example, sample (1000+250) contains 1000 mg of mouse feed slurry and 250 µL of the ^19^F MR tracer. All images were acquired by *T*_2_‐weighted ^1^H MR and ^19^F MR imaging with a 7T MRI scanner.

We measured the ^19^F MRI limit of detection of perfluorooctane and PDFEA_100_ (Figure 2D–F) by mixing various amounts of each tracer with various amounts of aqueous homogenized mouse feed slurry (Section S2.3.2). In all tracers, the limit of detection ranged from 60 to 120 µL of tracer per gram of chyme slurry (approx. 0.6 to 1.2 vol. %). Since perfluorooctane did not mix well with the chyme slurry, these samples provided a non‐homogeneous ^19^F MR signal, producing imaging artifacts [32]. In turn, PDFEA mixed well with the chyme model and provided a uniform signal throughout the sample. These results show that **PDFEA_100_** and **P(DFEA_63_‐*co*‐HPMA_37_)** have similar limits of detection to that of perfluorooctane, but mix well with the feed slurry, so they are more suitable tracers for GIT transit.

### 2.5 P(DFEA63‐co‐HPMA37) Yields a Strong ^19^F MR Signal for In Vivo GIT Transit Monitoring

Our proof‐of‐concept in vitro experiments highlighted the potential of hydrophilic polymers as ^19^F MRI tracers for GIT imaging, so we tested these compounds in an animal model. For animal testing, we used **P(DFEA_63_‐*co*‐HPMA_37_)** and **PDFEA_100_**. On the one hand, **P(DFEA_63_‐*co*‐HPMA_37_)** was chosen for its strong ^19^F MR signal, virtually unaffected by temperature and polymer dilution, two key properties for studying and quantifying GIT transitions. On the other hand, while **PDFEA_100_** was poorly soluble and lost approximately 50% of its ^19^F MR signal at 37°C, this polymer (a) aggregated into a gel, potentially filling the entire GIT, (b) its synthesis was simpler and less expensive, and (c) the residual 50% of MR signal should suffice for target applications. Moreover, (d) the aggregated polymer should function as a bulk‐forming agent without expanding the stool volume too much or inducing a laxative effect. To administer room‐temperature aqueous solutions of **P(DFEA_63_‐*co*‐HPMA_37_)** (2.5 ± 0.1 mL; 300 mg/mL; two rats), cool aqueous solutions of **PDFEA_100_** (2.5 ± 0.1 mL; 160 mg/mL; four rats), and saline (2.5 ± 0.1 mL; six rats), we used a gastric probe. Subsequently, we performed ^19^F MRS, ^1^H, and ^19^F MRI of rats in frequent random sampling for approximately 48 h (for details, see Section S3).

**P(DFEA_63_‐*co*‐HPMA_37_)** provided a strong ^19^F MR signal for detailed GIT transit monitoring (Figure 3). Shortly after administration, a ^19^F MRI signal was detected in the stomach and various segments of the small intestine (Figure 3). These ^19^F MR images showed that the polymer reached the ileum 80 min after administration and the caecum 6 h after administration. No ^19^F MR signal was detected in the stomach or ileum at 80 min and 6 h after administration, respectively. Approximately 25 h past administration, the polymer was almost fully excreted (residual barely‐detectable signal was found across the caecum; Figure 3, Figure S38, and Tables S6b and S7b) and the ^19^F MR signal was detectable in rat stool (Figure S37).

**FIGURE 3:**
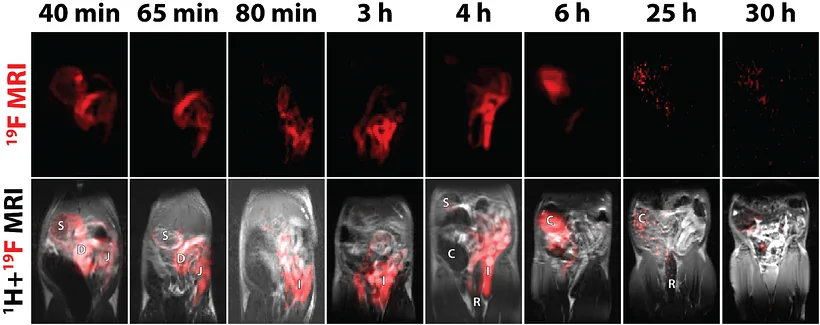
**P(DFEA_63_‐*co*‐HPMA_37_)** enables gastrointestinal (GIT) transit monitoring by fluorine‐19 MRI, frontal plane. Approximate locations of organs are depicted using ^1^H MRI and literature data [50]: stomach (S), duodenum (D), jejunum (J), ileum (I), caecum (C), and rectum (R). Due to the 15 mm slice thickness of the ^19^F MRI, these assignments of organs are indicative rather than definitive. The anatomy of the GIT is strongly affected by the position of the rat and the ^1^H MRI layer. All images were individually windowed to maximize the signal and minimize the noise. Residual low‐intensity ^19^F signal at 25 and 30 h colocalizes with the caecum and most likely reflects a small amount of residual polymer near the detection limit. Its patchy look is a display effect of per‐frame windowing of a near‐noise signal (Section S6.1 and Figure S38).

In contrast to **P(DFEA_63_‐*co*‐HPMA_37_)**, **PDFEA_100_** was not a reliable ^19^F MRI/ MRS tracer for GIT transit monitoring. Even though all four rats received identical amounts of the polymer and thus should have produced similar ^19^F MR signals, the signal varied greatly both among individual rats and over time (Figure S6). We attribute this ^19^F MR signal variability to thermoresponsiveness, as polymer aggregation in the GIT resulted in loss of ^19^F MR signal. Moreover, varying conditions (pH, polymer concentrations, and chyme) throughout the GIT may increase or decrease polymer aggregation, thereby inducing ^19^F MR signal variability. As only a few ^19^F MR images showed a detectable ^19^F signal (Figure S6), we concluded that **PDFEA_100_** is not a reliable tracer for GIT transit.

After administering **PDFEA_100_**, we did not observe any change in behavior or signs of maldigestion, constipation, or diarrhea. **P(DFEA_63_‐*co*‐HPMA_37_)** did increase defecation and softened the stool, which became slightly lighter in color, scoring 3 to 4 on the Bristol stool scale [51] (vs. 1 to 2 for **PDFEA_100_** and for the control without the polymer). This mild laxative effect has been reported for hydrophilic nonresorbable polymers, such as polyethylene glycol [52]. ^19^F MR imaging of stool from rats gavaged with **P(DFEA_63_‐*co*‐HPMA_37_)** showed that the polymer was not degraded or resorbed, given the strong signal (Figure S37). Control feces (without fluorinated polymer) did not yield any signal (data not shown). These results suggested that our polymers did not disturb GI function, which was confirmed by histopathological examination.

#### 2.5.1 Histopathological Examination Reveals No Pathology or Sign of Polymer Uptake

Following the in vivo experiments (24–48 h after administration), the rats were sacrificed for a thorough organ examination. We collected various organs: the stomach, large and small intestines, and cecum, as well as the kidneys, spleen, and liver, where parenterally administered polymers tend to accumulate [53, 54, 55, 56, 57, 58, 59, 60], so they are the most sensitive markers of polymer absorption and toxicity. One rat (P3) had melaena in its gut and a stomach injury, likely caused by the rigid GIT feeding probe. Apart from this single anomaly, all organs seemed macroscopically healthy, with no other sign of damage. The remaining organs did not show any sign of macroscopic or microscopic pathology (Figure 4); we observed only a slightly increased accumulation of lymphatic tissue in the GIT, particularly in the cecum. Overall, our histopathological and organ morphology analysis showed no sign of toxicity, confirming that **PDFEA** is biologically well tolerated and suitable for GIT examination by ^19^F MRI. The polymers did not accumulate in organs outside the GIT (Figures S34–S36), corroborating the findings of studies on polymers with negligible‐to‐null bioavailability from the GIT [61, 62, 63, 64, 65, 66]. Therefore, **PDFEA** tracers are biologically safe, nonresorbable, and well‐suited for GIT transit monitoring.

**FIGURE 4:**
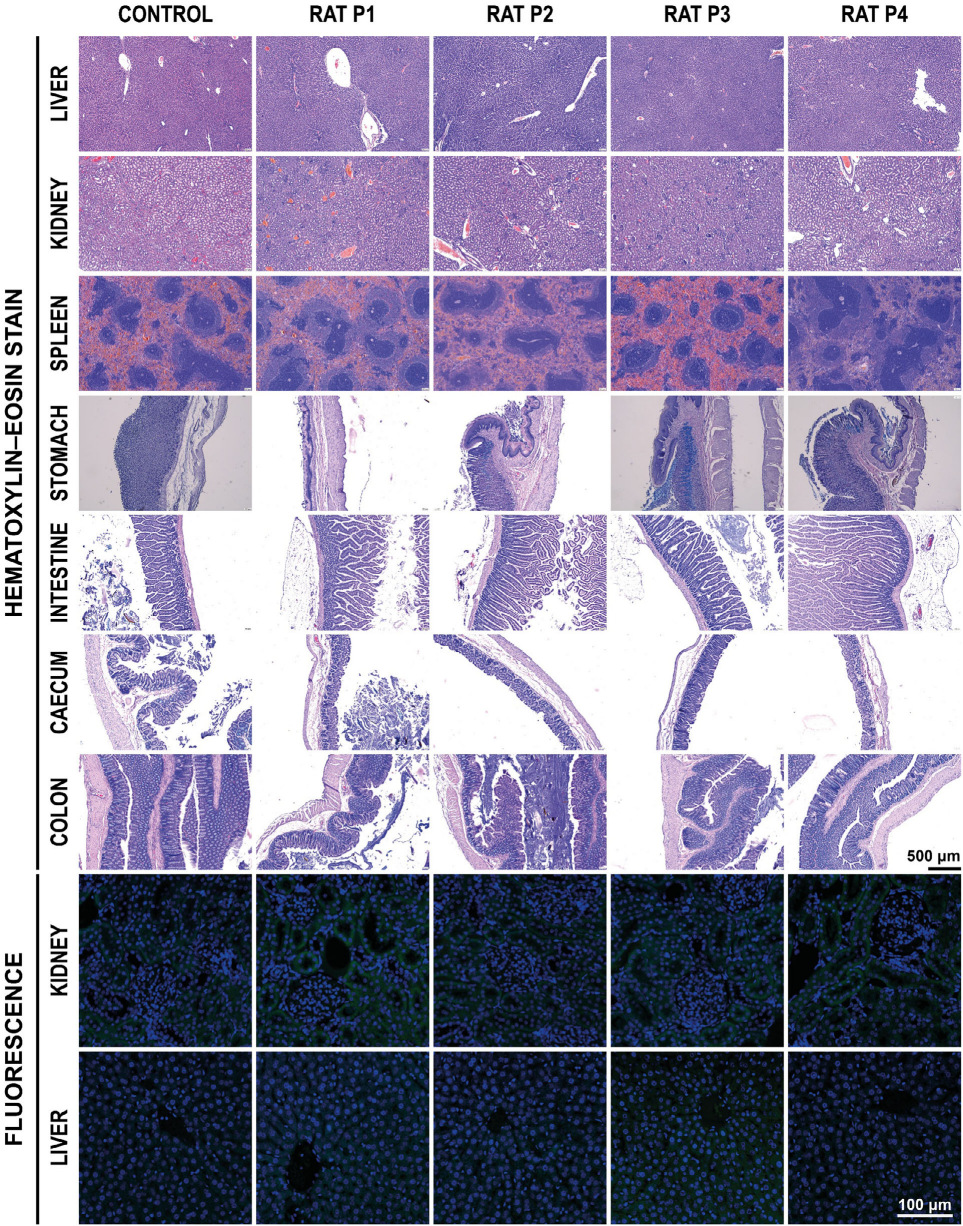
Histopathological examinations of organs of control and P1 to P4 rats, 24–48 h after polymer administration, show that our polymers are biologically inert (hematoxylin‐eosin images) and non‐resorbable (fluorescence images). All samples were deemed physiological; the differences between images were attributed to sampling or staining variations. Fluorescence samples were stained with DAPI (nuclear stain, blue), and the fluorescein signal is shown in green. Apart from mild naturally occurring tissue autofluorescence, no fluorescence was detected, suggesting only negligible‐to‐null uptake of study polymers from GIT.

## 3 Discussion

In this study, we developed cost‐effective, hydrophilic fluorinated polymer ^19^F MRI tracers for GIT transit monitoring (Figure 1) with suitable ^19^F MR properties (Figure 2 and Table S8). These tracers mixed well (Figure 2) and moved (Figure 3) with chyme along the GIT over time and proved non‐resorbable, biologically inert, and non‐toxic (Figure 4). Although designing hydrophilic, non‐resorbable compounds can be challenging [67], hydrophilic polymers show low‐to‐negligible GIT resorption when their molar mass is high [61, 62, 63, 64, 65, 66]. However, all polymer samples with a dispersity index (*Đ*_M_) > 1 contain polymer chains with various lengths, and short hydrophilic polymer chains may be absorbed from the GIT [64]. To minimize the content of short polymer chains, we prepared polymers with a relatively high *M*_W_ (≈ 50 kg/mol) and with the lowest possible *Đ*_M_ to prevent systemic uptake. As such, our polymers can still be easily excreted as non‐metabolized polymer chains through the kidneys [53, 68], even if some of their chains are absorbed due to GIT immaturity [69, 70], GIT diseases [64], or liver [63] diseases (further discussed in section S1).

Initially, **PDFEA_100_** was tested for ^19^F MR imaging because this polymer had the highest fluorine content per gram of dry polymer (28.4 wt. %, Figure 1B). But its tendency to aggregate at body temperature decreased the *T*_2_ relaxation of a portion of ^19^F atoms to markedly low values (<1 ms), rendering them invisible to ^19^F MRI [44] (Figure 1D). In line with the findings of our previous studies [44, 45], this result demonstrated that the ^19^F MR signal of PDFEA weakens at higher temperatures due to polymer aggregation. Even subtle changes in conditions, such as temperature, concentration (Figure 1D), ions, and pH [71], and in food precipitated bidirectional fluctuations in ^19^F MR intensity, yielding a poor ^19^F MR tracer. In turn, **P(DFEA‐*co*‐HPMA)** copolymers had a lower fluorine content per gram of pure polymer than **PDFEA_100_**, but they were more soluble in water, so they reached higher overall ^19^F concentrations and, as a result, produced a stronger ^19^F MR signal. Moreover, they were much less susceptible to aggregation and signal fluctuations. These properties explain why **P(DFEA‐*co*‐HPMA)** are more robust ^19^F MR tracers than **PDFEA_100_** (Figure 1D).

Hydrophilic **P(DFEA‐*co*‐HPMA)** copolymers also offer two main advantages over previously explored perfluorocarbons [28, 29, 30, 37, 38]. First, **PDFEA** provides a single, sharp ^19^F MR signal with suitable MR relaxation properties, whereas perfluorocarbons usually contain multiple ^19^F MR signals with poor ^19^F MR relaxation properties for common imaging sequences (Table S8). Second, **P(DFEA‐*co*‐HPMA)** copolymers mix well with chyme (Figure 2), unlike fluorocarbons, which are both hydro‐ and lipophobic, causing imaging artifacts (intraluminal layering [32] and gastric sieving [72]). For all these reasons, we deem **P(DFEA‐*co*‐HPMA)** copolymers as a more reliable and versatile platform for ^19^F MR GIT tracers.

After administering **P(DFEA_63_‐*co*‐HPMA_37_)** into rats, we observed a strong ^19^F MR signal moving through their GIT (Figure 3). By ^1^H+^19^F MRI, we were able to approximately identify the anatomical location of the polymer at various time points. In addition, we did not observe any change in animal behavior or signs of toxicity, maldigestion, constipation, or diarrhea other than a mild stool‐softening effect. While this softening effect may alter the transit times over the native state, we expect the softening to shift healthy and pathological subjects by a comparable margin, preserving the relative difference between the two groups and thus the diagnostic power of the measurement. In line with that, the currently approved imaging agents also alter the transit times (barium sulphate frequently causes constipation, and mannitol‐based bowel MRI preparations exert a pronounced osmotic laxative effect) and are still diagnostically useful. Such mild side effects are acceptable for clinical applications and do not deteriorate the diagnostic power of tracing agents. Our behavioral findings were confirmed by a meticulous histopathological examination, which did not reveal any sign of toxicity to organs either (Figure 4). **P(DFEA_63_‐*co*‐HPMA_37_)** copolymers also proved non‐resorbable when labelled with fluorescein, with no accumulation in the liver or kidneys (Figure 4). Overall, our results show that **P(DFEA_63_‐*co*‐HPMA_37_)** is a safe, well‐tolerated, effective, reliable, and non‐resorbable ^19^F MR tracer.

Our proof‐of‐concept in vivo experiments show that the polymer can be tracked through the rat GIT by ^19^F MRI, although the model imposed several constraints that will not apply in human subjects. The ^19^F MR tracer took approximately 6 h to reach the colon of the rats (Figure 3). However, feed should reach the rat colon within 1 to 2 h from administration [28, 73, 74, 75]. This discrepancy may be ascribed to differences in feed composition [76, 77] and anesthesia [78], which are known to alter GIT motility. During our study, our rats remained under anesthesia 50% of the time, which slowed their transit significantly [78]. In contrast, human patients will not require anesthesia for imaging, as they can simply drink the polymer solution and the ^1^H+^19^F MRI can be acquired in their conscious state. Moreover, the rat models were administered with mere ≈ 750 mg of polymers, which limited the ^19^F MR signal and forced us to use large voxels, diminishing the imaging resolution. In turn, human subjects may easily receive ≥ 100 grams of polymer, thereby increasing ^19^F signal, shortening the MR acquisition times, and increasing the ^19^F MRI resolution. Despite these experimental constraints, the polymer was readily detectable and trackable throughout the rat GIT. In human subjects, where none of these limitations apply, ^1^H+^19^F MRI coupled with our tracers should provide substantially superior transit data.

Notwithstanding their performance as ^19^F MRI tracers, these polymers contain amide moieties that may be partly hydrolyzed under gastric conditions, releasing amines, which may cause side effects. Nevertheless, amides are fairly resilient to hydrolysis [83], and substituted poly(acrylamides) are only slowly hydrolyzed under extremely acidic and basic conditions (aqueous 0.5 M NaOH or pH ≤1) and over long periods (>120 h) [84], well above stomach dwelling times [74]. If desired, the tracer could also be co‐administered with antacids or formulated with an enteric coating to bypass gastric exposure entirely. To offset the thermoresponsiveness of **PDFEA**, we incorporated HPMA, but other hydrophilic co‐monomers may yield even better tracers. Non‐thermoresponsive fluorinated polymers, such as poly(2‐fluoroethylacrylamide) (**PFEA**) [85], may also be tested, as long as their ^19^F content is high enough for in vivo imaging. Additionally, hydrophilic polymers may be further functionalized with fluorinated polymers that are known to change their ^19^F MR chemical shift as a function of pH, ion concentration, oxidative conditions, and enzymatic activity [86] to track local changes in intestinal function and anatomy. Further research should also be conducted to test these ^19^F MRI tracers in disease models and humans to assess their safety and efficacy, especially in GIT diseases.

For now, ^1^H+^19^F MRI coupled with **P(DFEA‐*co*‐PHPMA)** offers the first reliable, repeatable, radiation‐free, and noninvasive method for assessing both GIT transit time (after a single bolus administration) and GIT anatomy (continuous administration). Considering its potential applications to routine clinical practice, this cost‐effective method may complement or replace scintigraphy and contrast radiography in the evaluation of GIT motor function. Since it avoids ionizing radiation [14], GIT transit can be assessed repeatedly in a wide range of patient populations (including children) for both diagnosis and treatment monitoring — particularly where cumulative radiation exposure from repeated radiographic transit studies is a serious concern. With functional GIT diseases (gastroparesis, chronic constipation, functional diarrhea, etc.) affecting approximately 40% of the global population [7], this much‐needed method may propel research and provide patients with better treatment options [8, 9, 87, 88, 89]. Organic GIT diseases may also benefit from ^1^H+^19^F MRI GIT tracers. By 2040, nearly 1.5% of the human population is projected to suffer from inflammatory bowel disease [90]. These conditions require routine check‐ups to detect fistulas and obstructions; high‐resolution ^1^H images with overlaid ^19^F signal may enable non‐invasive detection of obstructions, polyps, diverticula, and other structural abnormalities, without the long preparation phase or dietary restrictions of colonoscopy (a ^19^F MR analogue of 3D virtual colonoscopy). Such a safe and quantitative GIT transit examination could become a benchmark tool in gastroenterological drug development by enabling a systematic evaluation of both desired and off‐target effects of novel intestinal motility therapies. Ultimately, ^1^H+^19^F MRI with GIT tracers will expand the palette of clinical and research tools.

## 4 Conclusion

Hydrophilic fluorinated polymers enable GIT transit monitoring by ^19^F MRI. These polymers in combination with ^1^H+^19^F MRI provide an alternative to invasive methods and radiographical techniques, such as irrigography, defecography and contrast radiography. PDFEA copolymers surpass the physicochemical (high water solubility) and ^19^F MR (more suitable relaxation times, single ^19^F MR chemical shift) properties of fluorocarbons, producing a homogeneous ^19^F MR signal throughout the chyme and avoiding imaging artifacts. In particular, **P(DFEA_63_‐*co*‐HPMA_37_)** is a safe, well‐tolerated, effective, reliable, and nonresorbable ^19^F MR tracer for the GIT that delivers accurate GIT anatomy and transit data. Further research should be conducted to test these ^19^F MRI tracers in disease models and humans to assess their safety and efficacy.

## Author Contributions

**Filip Koucký**: investigation, methodology, validation, formal analysis, data curation. **Zuzana Pavlíková**: investigation, methodology, validation, formal analysis, data curation. **Margarita Tkachenko**: investigation, validation, methodology, formal analysis, data curation. **Mária Hovořáková**: investigation, methodology, validation, formal analysis, data curation. **Klára Steklíková**: investigation, methodology, validation, formal analysis, data curation. **Linda Dalecká**: investigation, methodology, validation, formal analysis, data curation. **Petr Páral**: investigation, validation, methodology, formal analysis, data curation. **Petr Matouš**: investigation, methodology, validation, formal analysis, data curation. **Jiří Beneš**: writing – review and editing, funding acquisition, supervision, resources, validation. **Ondřej Groborz**: conceptualization, investigation, funding acquisition, writing – original draft, methodology, validation, visualization, formal analysis, project administration, data curation, supervision. **Ludmila Maffei Svobodová**: investigation, validation, methodology, formal analysis, data curation. **Kristýna Kolouchová**: resources, supervision, project administration, data curation, formal analysis, methodology, validation, writing – review and editing, funding acquisition, investigation. **Vít Herynek**: methodology, validation, formal analysis, project administration, resources, supervision, data curation, software, investigation. **Fatma Nalan Cetin**: investigation, validation, methodology, formal analysis, data curation.

## Funding

The authors thank Dr. Carlos V. Melo for editing the manuscript, Professor Sandra van Vlierberghe, David Dunlop, and Professor Tomáš Slanina for their insightful comments and guidance during the project and manuscript writing, and Charles University Grant Agency (GAUK) for funding this research, under project No. 199124. K.K. and F.N.C. gratefully acknowledge the financial support provided by the Research Foundation – Flanders (FWO, Fonds Wetenschappelijk Onderzoek–Vlaanderen, project No. 1247425N, G075621N, 1SC1225N) and the COOPERATIO Program of Charles University, Prague, Czech Republic (207036‐10 Morphological Disciplines of Medicine/LF1). The authors also thank the core facility Center for Advanced Preclinical Imaging, Charles University, supported by the MEYS CR (LM2023050 Czech‐BioImaging), by the European Regional Development Fund (Project No. CZ.02.1.01/0.0/0.0/18_046/0016045), and by Charles University grant SVV 260 635/2025. Computational resources were provided by the e‐INFRA CZ project (ID:90254), supported by the Ministry of Education, Youth and Sports of the Czech Republic.

## Conflicts of Interest

The authors declare no conflicts of interest.

## Supporting information




**Supporting File**: adhm71667‐sup‐0001‐SuppMat.docx.

## Data Availability

The data that support the findings of this study are openly available in Polymers for Gastrointestinal Transit Monitoring via 19‐F MR at osf.io/c4xr7.
